# Co-Administration of Resveratrol and Lipoic Acid, or Their Synthetic Combination, Enhances Neuroprotection in a Rat Model of Ischemia/Reperfusion

**DOI:** 10.1371/journal.pone.0087865

**Published:** 2014-01-31

**Authors:** Monique C. Saleh, Barry J. Connell, Desikan Rajagopal, Bobby V. Khan, Alaa S. Abd-El-Aziz, Inan Kucukkaya, Tarek M. Saleh

**Affiliations:** 1 Department of Biomedical Sciences, Atlantic Veterinary College, University of Prince Edward Island, Charlottetown, P.E.I., Canada; 2 Department of Chemistry, University of Prince Edward Island, Charlottetown, P.E.I., Canada; 3 Carmel BioSciences Inc., Atlanta, Georgia, United States of America; School of Pharmacy, Texas Tech University HSC, United States of America

## Abstract

The present study demonstrates the benefits of combinatorial antioxidant therapy in the treatment of ischemic stroke. Male Sprague-Dawley rats were anaesthetised and the middle cerebral artery (MCA) was occluded for 30 minutes followed by 5.5 hours of reperfusion. Pretreatment with resveratrol 30 minutes prior to MCA occlusion resulted in a significant, dose-dependent decrease in infarct volume (p<0.05) compared to vehicle-treated animals. Neuroprotection was also observed when resveratrol (2×10^−3 ^mg/kg; iv) was administered within 60 minutes following the return of blood flow (reperfusion). Pretreatment with non-neuroprotective doses of resveratrol (2×10^−6^ mg/kg) and lipoic acid (LA; 0.005 mg/kg) in combination produced significant neuroprotection as well. This neuroprotection was also observed when resveratrol and LA were administered 15 minutes following the onset of MCA occlusion. Subsequently, we synthetically combined resveratrol and LA in both a 1∶3 (UPEI-200) and 1∶1 (UPEI-201) ratio, and screened these new chemical entities in both permanent and transient ischemia models. UPEI-200 was ineffective, while UPEI-201 demonstrated significant, dose-dependent neuroprotection. These results demonstrate that combining subthreshold doses of resveratrol and LA prior to ischemia-reperfusion can provide significant neuroprotection likely resulting from concurrent effects on multiple pathways. The additional protection observed in the novel compound UPEI 201 may present opportunities for addressing ischemia-induced damage in patients presenting with transient ischemic episodes.

## Introduction

Despite ongoing advances in the arena of stroke research, the worldwide consequences of death and disability remain considerable and delivery of successful therapeutics continues to present a challenge. The application of combinatorial drug therapy in treating stroke has become increasingly attractive in recent years. As researchers uncover the complexity of disease progression following stroke which includes both immediate as well as delayed neuronal effects at multiple levels [1], it has become evident that multi-targeted drug therapy may hold more promise in the treatment and/or prevention of stroke than conventional single class drug regimens. In addition, there is evidence that some drug combinations display pharmacological potentiation (ie synergism) which optimistically translates into lower doses, fewer adverse side effects and an extended treatment window. Treatment outcomes for ischemic events involves the reestablishment of blood flow to compromised tissue, with the reintroduction of oxygen transiently adding to the injury due to generation of inflammatory mediators and toxic levels of oxidative free radicals [2] culminating in lipid peroxidation, protein synthesis arrest, and ultimately cell death [3]. Successful treatment options are therefore required to address several critical mediators of neuronal death simultaneously.

With the increasing popularity of natural products, science has sought to exploit the medicinal potential of common extracts as is evident in the growing literature of natural product drug discovery. In the current study, we test 2 novel compounds combining resveratrol (3, 5, 4′-trihydroxystilbene), a naturally-occurring component of grapes, and α-lipoic acid (LA), a potent anti-oxidant found in common foods, for neuroprotective effects in 2 animal models of ischemic stroke. Separately, resveratrol and LA possess potent anti-oxidant and anti-inflammatory activities and have been shown to produce neuroprotection in several animal models of neurological disease via complementary pathways [4] [5].

Resveratrol possesses multiple biological activities [6] [7], including being a potent antioxidant [8] and anti-inflammatory [9] agent. These therapeutic uses of resveratrol have led researchers to investigate its protective effects in several animal models of neurological disease, particularly those with unknown etiology, or where inflammation and oxidative stress may play a role in the pathogenesis. Consequently, resveratrol has shown promise as a neuroprotectant in animal models of cerebral ischemia through its ability to attenuate ischemia-induced cell death [10].

Also a powerful antioxidant, α-lipoic acid (LA) is characterized by high reactivity towards free radicals [11] and demonstrates potent neuroprotective effects in several animal models of stroke including models of reperfusion injury [12] [13] [14] [15] [16] [17]. Further, the co-administration of LA with other compounds has been shown to enhance the protective effect of the drug in various animal models of pathology [18] [19] [20] [21] [22]. Our own research has shown that co-administration of non-protective (sub-threshold) doses of both LA and apocynin, an NADPH oxidase inhibitor, provided significant neuroprotection against ischemic injury [23].

Thus, the current study investigated the potential for enhanced neuroprotective effects with LA by combining it with resveratrol in a rodent model of acute stroke and reperfusion injury [24]. In addition, the effects of resveratrol and lipoic acid were compared to UPEI-200 and UPEI-201, two novel synthetic compounds linking resveratrol with LA in both a transient occlusion-reperfusion model (tMCAO) as well as in a permanent occlusion model (pMCAO). Lastly, the feasibility of delayed treatment intervention was investigated for all drug combinations.

## Methods

### Ethics Statement

All experiments were carried out in accordance with the guidelines of the Canadian Council on Animal Care and were approved by the University of Prince Edward Island Animal Care Committee (protocol #11-045 and 13-036).

### General Surgical Procedures for in vivo Studies

All experiments were conducted on male Sprague-Dawley rats (250–350 g; Charles Rivers; Montreal, PQ, CAN). For all animals, food and tap water were available *ad libitum*. Rats were anaesthetized with sodium thiobutabarbital (Inactin; Sigma-Aldridge; St.Louis, MO, USA; 100 mg/kg; ip) and supplemented as needed. For intravenous administration of drugs, a polyethylene catheter (PE-10; Clay Adams, Parsippany, NJ, USA) was inserted into the right femoral vein. An endotracheal tube was inserted to facilitate breathing. Body temperature was monitored and maintained at 37±1°C using a feedback system (Physitemp Instruments; Clifton, NJ, USA).

A separate group of animals were instrumented for the recording of blood pressure and heart rate via an indwelling catheter (PE-50; ; Clay Adams, Parsippany, NJ, USA) placed inside the right femoral artery. Arterial blood pressure was measured with a pressure transducer (Gould P23 ID, Cleveland, OH) connected to a Gould model 2200S polygraph. Heart rate was determined from the pulse pressure using a Gould tachograph (Biotach). These parameters were displayed and analyzed using PolyviewPro/32 data acquisition and analysis software (Grass Technologies, Warwick, RI).

### Transient and Permanent Middle Cerebral Artery Occlusions (tMCAO and pMCAO)

We have previously published the detailed methodology for transient occlusion of the middle cerebral artery [24]. Briefly, animals were placed in a David Kopf stereotaxic frame (Tujunga, CA, USA) and the right middle cerebral artery (MCA) approached through a rostra-caudal incision of the skin and frontalis muscle at the approximate level of bregma. Blood flow through the MCA was impeded by the placement of surgical suture behind the MCA at 3 designated positions along the exposed vessel for 30 minutes in the transient model (tMCAO), or left in place for a total of 6 hours in the permanent model (pMCAO). The sutures were positioned such that the middle of each suture applied pressure to the underside of the MCA and impeded blood flow (ischemia) as previously confirmed using laser Doppler flowmetry (OxyFlo, Oxford-Optronix, Oxford, UK) (Connell and Saleh 2010). This 3-point placement of surgical sutures produced a highly reproducible and consistent focal ischemic lesion restricted to the prefrontal cerebral cortex. Blood flow in the tMCAO model was re-established (reperfusion) for an additional 5.5 hours following removal of the sutures.

### Drug Preparation

Resveratrol (trans-3,5,4′-trihydroxy stilbene; Sigma Aldrich, St. Louis, MO, USA) stock solutions were prepared in 40% propylene glycol and diluted 10,000X in 0.9% saline The concentration of propylene glycol in each solution was 4×10^−3%^ (v/v). Lipoic acid (LA; Sigma-Aldridge; St. Louis, MO, USA; 0.005 mg/ml) was prepared in physiological saline (0.9% sodium chloride) and the pH was adjusted to 7.0–7.4 with sodium hydroxide. The concentration of LA used was previously determined to be non-neuroprotective in our tMCAO model [12]. Appropriate vehicle solutions were prepared for each drug and dose.

### Synthesis of UPEI-200

The chemical synthesis of UPEI-200 was performed as follows; resveratrol (0.01 M) was combined with 0.05 M LA and 0.04 M of dimethylaminopyridine (DMAP) in 80 ml of anhydrous dichloromethane (CH_2_Cl_2_). 1-Ethyl-3-(3-dimethylaminopropyl) carbodimide hydrochloride (EDCI; 0.05 M) was added in small quantities over a period of 2 hours. The entire reaction was performed under nitrogen atmosphere at room temperature. After stirring overnight, the crude mixture of compounds was quickly purified by passing through a silica column following an aqueous work up. The product was again purified on a Chromatotran silica plate using 2 mm pre-coated UV active plate. Appropriate fractions were mixed and concentrated in a rotary evaporator keeping the water bath temperature at 45°C. The final pure compound was obtained as a pale yellow viscous solid in very low yield and was characterized by proton nuclear magnetic resonance spectroscopy and mass spectrometry.

### Synthesis of UPEI-201

Resveratrol (1 mmol) in 20 ml dimethylformamide (DMF) was combined with DMAP (10 mmol) and LA (1 mmol). Dicyclohexylcarbodimide (DCC; 1 mmol) was added to the reaction mixture at 0°C under nitrogen atmosphere, which is then stirred for 5 min at 0°C and 3 h at 20°C. Precipitated urea is then filtered off and the filtrate evaporated down *in vacuo*. The residue was taken up in dichloromethane (CH_2_Cl_2_) and, if necessary, filtered free of any further precipitated urea. The solvent was removed by evaporation and the crude compound was purified by silica column chromatography (Eluent, Hexanes:Ethylacetate (1∶1; yellow oil), Yield; 57%.^1^H NMR (300 MHz, Acetone) δ 7.40 (2H, d, *J* = 8.6 Hz), 7.05–6.79 (4H,m), 6.52 (2H, d, *J* = 2.1 Hz), 6.25 (1H, t, *J* = 2.1 Hz), 3.58 (1H, tt, *J* = 12.7, 6.4 Hz), 3.24–3.04 (2H, m), 2.45 (1H, tt, *J* = 12.3, 6.2 Hz), 2.28 (1H, t, *J* = 7.2 Hz), 1.95–1.80 (1H, m), 1.79–1.52 (4H, m), 1.45 (2H, dtd, *J* = 11.1, 7.1, 4.1 Hz).

### Effect of Resveratrol on tMCAO Model

In the first experiment, resveratrol (2×10^−3^ (n = 5), 2×10^−4^ (n = 5), 2×10^−5^ (n = 6), 2×10^−6^ (n = 5), 2×10^−7^ (n = 5) mg/kg; 1 ml/kg; i.v.) or vehicle (propylene glycol; 4×10^−3%^ (v/v); 1 ml/kg; i.v.; n = 5) was administered 30 minutes prior to the onset of MCAO. The sutures were left in place for 30 minutes, followed by 5.5 hours of reperfusion.

The feasibility of extended treatment options was investigated by administering the highest dose of resveratrol (2×10^−3^ mg/kg; i.v.) or vehicle (propylene glycol; 4×10^−3%^ (v/v); 1 ml/kg; i.v.) at the following intervals during the I/R protocol; 15 minutes (n = 5/group) following the onset of MCAO, and 0, 30, 60, 90 minutes (n = 5,6,7,4 respectively) following the onset of reperfusion.

### Co-administration of Resveratrol and Lipoic Acid (tMCAO)

To examine neuroprotection following co-administration of various doses of resveratrol (2×10^−5^ (n = 5), 2×10^−6^ (n = 5), 2×10^−7^ (n = 6), 2×10^−8^ (n = 6), or 2×10^−9^ (n = 6) mg/kg) with LA (0.005 mg/kg) on ischemia-reperfusion injury in our tMCAO model, resveratrol and LA were combined into a single solution and administered (1.0 ml/kg; iv) 30 minutes prior to MCAO. The MCA was occluded for 30 minutes followed by 5.5 hours of reperfusion.

Delayed treatment effects were also studied by co-injecting resveratrol (2×10^−5^ mg/kg) and LA (0.005 mg/kg; i.v) at the following intervals during the I/R protocol; 15 minutes (n = 8) following the onset of MCAO, and 0, 30, 60, 90 minutes (n = 6,6,7,4 respectively) following the onset of reperfusion.

### Co-administration of Resveratrol and Lipoic Acid and Permanent Occlusion (pMCAO)

To determine if the co-administration of resveratrol and LA was neuroprotective on ischemia-induced cell death only, co-injection of resveratrol and LA (2×10^−5^ mg/kg and LA, 0.005 mg/kg; i.v.; n = 4) or vehicle (propylene glycol; 4×10^−3%^ (v/v); 1 ml/kg; i.v.; n = 4) were made 30 minutes prior to pMCAO. The experiments were terminated at the end of 6 hours of occlusion with no reperfusion period.

### UPEI-200 or 201 Effects in tMCAO or pMCAO

The effects of UPEI-200 and UPEI-201 on infarct volume in both transient and permanent MCAO models were investigated. Dose-response curves were generated for both entities (n = 4–7/group). UPEI-201 was further studied for its effectiveness in delayed intervention by administering a neuroprotective dose (1×10^−6^ mg/kg) 15 minutes post-occlusion as well as 0, 30 and 60 minutes into the 5.5 hr reperfusion period (n = 4–7/group).

### Histological Procedures

At the end of each experiment, in which infarct volume was measured, animals were transcardially perfused with phosphate buffered saline (PBS; 0.1 M; 200 mL). The brains were removed and sliced into 1 mm coronal sections with the aid of a rat brain matrix (Harvard Apparatus; Holliston, MA, USA). Sections were incubated in a 2% solution of 2,3,5-triphenol tetrazolium chloride (TTC; Sigma-Aldrich; St. Louis; MO, USA) for 5 minutes. Infarct volumes were calculated with measurements taken from scanned digital images of each brain section. The infarct area for opposing views of each brain section was calculated using a computer-assisted imaging system (Scion Corporation; Frederick, MD, USA), averaged and multiplied by section thickness (1 mm) to give a measure of infarct volume for each section. The sum total of the individual infarct volumes provided the infarct volume for each rat.

### Co-administration of Resveratrol - Lipoic Acid and Apoptosis

In a separate set of experiments, the co-administration of resveratrol (2×10^−5^ mg/kg) and LA (0.005 mg/kg; i.v.; n = 4) or vehicle (propylene glycol; 4×10^−3%^ (v/v); 1 ml/kg; i.v.; n = 4) were made 30 minutes prior to tMCAO. The sutures were left in place for 30 minutes followed by 5.5 hours of reperfusion. Animals were transcardially perfused with 200 mL of 0.1 M phosphate buffered saline (pH 7.4), the brains removed and the ipsilateral cerebral cortex isolated by careful dissection. A biopsy needle having an internal diameter of 8 mm was used to collect tissue from the region of infarct. The region of infarct was visually identified as that area which displayed a grayish hue and was slightly swollen compared to the surrounding healthy tissue. The biopsy needle was centered on this area and the tissue sample removed.

The tissue was weighed and homogenized (20% w/v) in ice cold PBS. The homogenate was centrifuged 12 000×g for 15 min at 4° C. Aliquots of the supernatant were stored at −80°C until assayed for protein. Apoptotic cell death was quantified using an ELISA based assay for determination of cytoplasmic histone-associated DNA fragments (Roche Diagnostics, Montreal, QC, CAN).

### Statistical Analysis

Data were analyzed using a statistical software package (SigmaStat and SigmaPlot; Jandel Scientific, Tujunga, CA). All data are presented as a mean ± standard error of the mean (S.E.M). Differences were considered statistically significant if p≤0.05 by an analysis of variance (ANOVA) followed by a Bonferroni post-hoc analysis. When only two groups were being compared the Student’s t-test was used.

## Results

### Resveratrol and tMCAO

**Figure 1:**
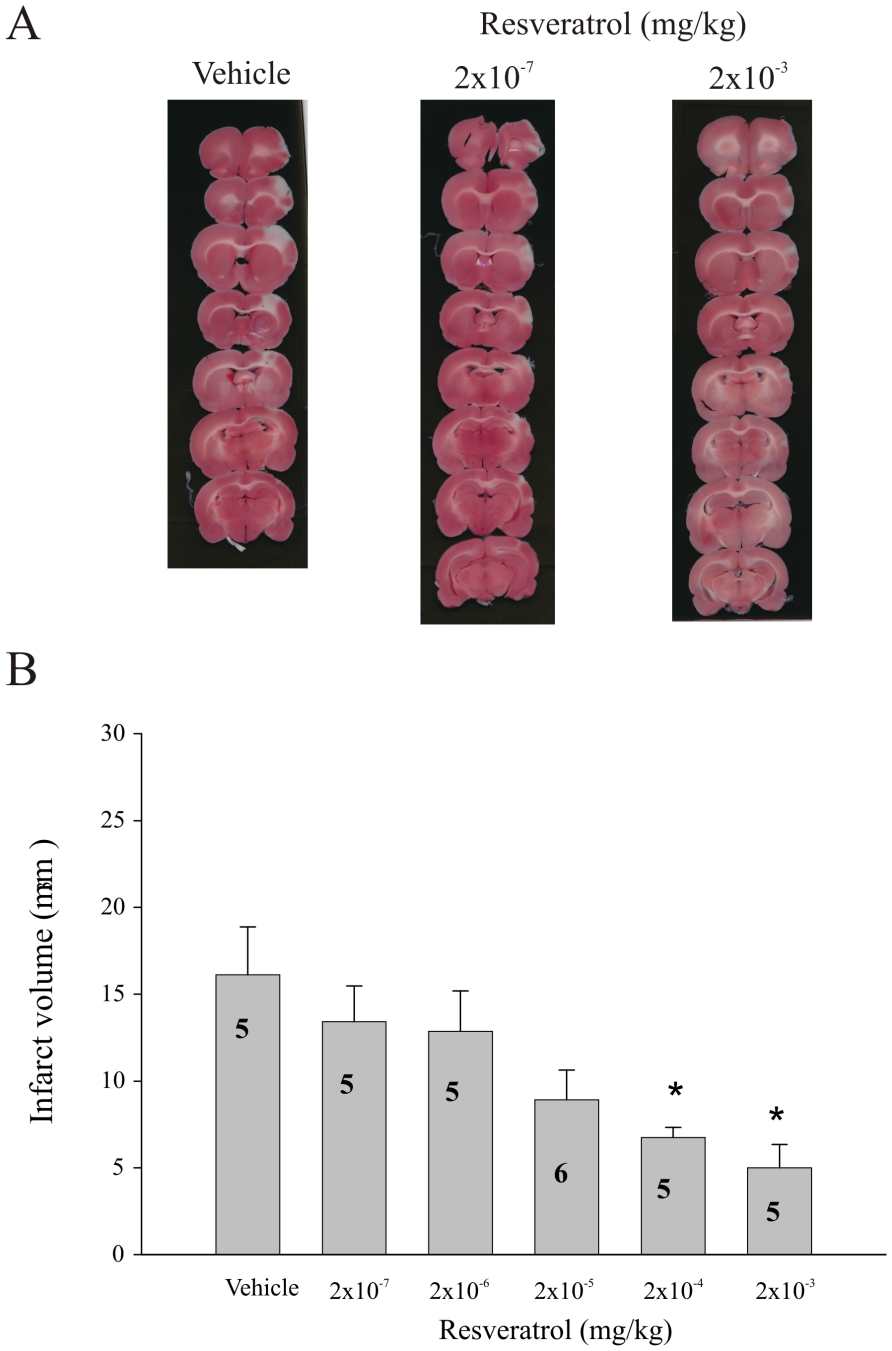
Dose-dependent effect of resveratrol on infarct volume following transient ischemia/reperfusion. (A) Representative photomicrographs of TTC-stained, 1 mm thick coronal slices illustrating the extent of infarct within the prefrontal cortex following pretreatment (30 minutes prior to MCAO; i.v.) with either Vehicle (propylene glycol 4×10^−3%^ (v/v)) or Resveratrol (2×10^−7^ and 2×10^−3 ^mg/kg) following ischemia/reperfusion (tMCAO). (B) Bar graph summarizing the dose-response relationship between increasing doses of resveratrol and infarct size (mm^3^) calculated from TTC-stained, 1 mm thick coronal sections following tMCAO. Each bar represents the mean ± S.E.M. (n = 5–6/group) and * indicates significance (*p*≤0.05) from the vehicle-treated control group.

Pre-administration of resveratrol provided dose-dependent neuroprotection in our model of ischemia-reperfusion. This was evident by a reduction in mean infarct volume with increasing doses of resveratrol (Figs. 1A and B). A significant difference in infarct volume between resveratrol treated animals and vehicle treated controls was observed at the 2 highest doses tested (2×10^−3^ and 2×10^−4^ mg/kg; p≤0.05).

**Figure 2:**
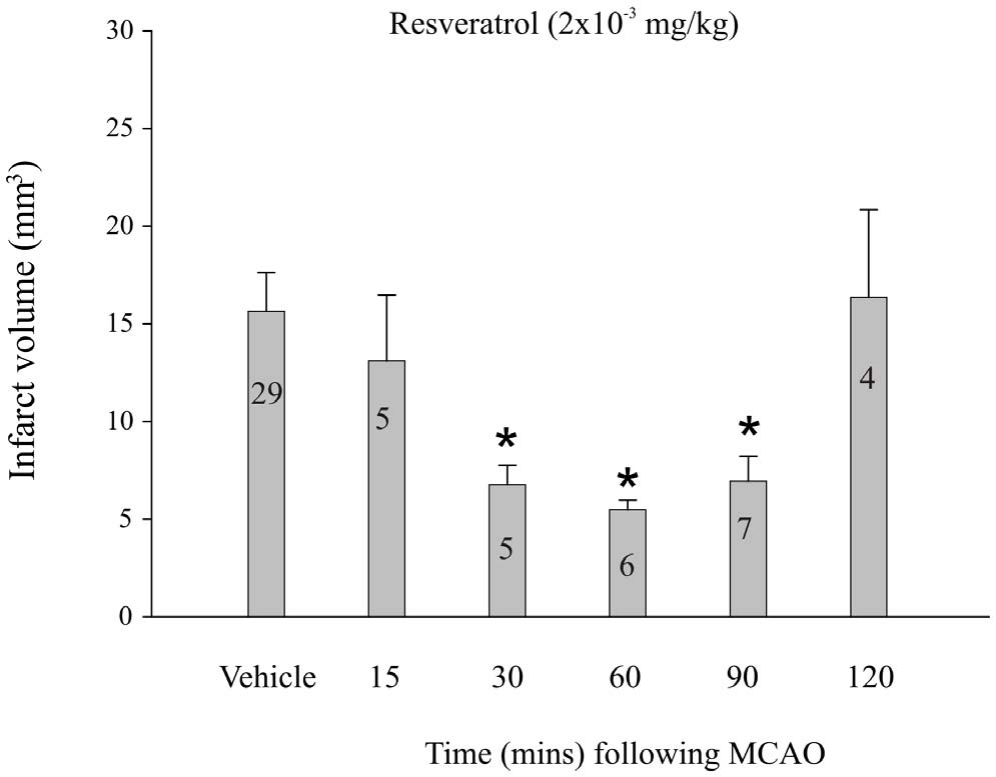
Time course of resveratrol-induced neuroprotection. Bar graph summarizing the effect of resveratrol injected 15 minutes into middle cerebral artery occlusion (15), or at 30 min intervals post-reperfusion. Each bar represents the mean ± S.E.M. (n = 5–6/group) and * indicates significance (*p*≤0.05) from the pooled vehicle-treated (propylene glycol 4×10^−3%^ (v/v)) control group.

Resveratrol or vehicle was injected during MCAO or during the period of reperfusion (Figure 2). There were no significant differences in the mean infarct volumes when vehicle was injected during MCAO or at any time point during reperfusion (p≥0.05), therefore, the vehicle data for all time points was pooled (n = 29). However, all statistical comparisons were made between the infarct volumes measured following resveratrol and vehicle administration for each time point. When resveratrol treatment (2×10^−3^ mg/kg; i.v.) was delayed until 15 minutes into the ischemic period or 90 minutes into the reperfusion period (120 min post occlusion) there was no effect on infarct volume when compared to vehicle injected controls (p≥0.05; Fig. 2). However, significant neuroprotection was observed when resveratrol (2×10^−3^ mg/kg) was administered at the start of the reperfusion period (30 minutes post-occlusion), or at 30 and 60 minutes into the reperfusion period (60 and 90 minutes post-occlusion; p≤0.05 at each time point; Fig. 2).

### Co-administration of Resveratrol and Lipoic Acid

**Figure 3:**
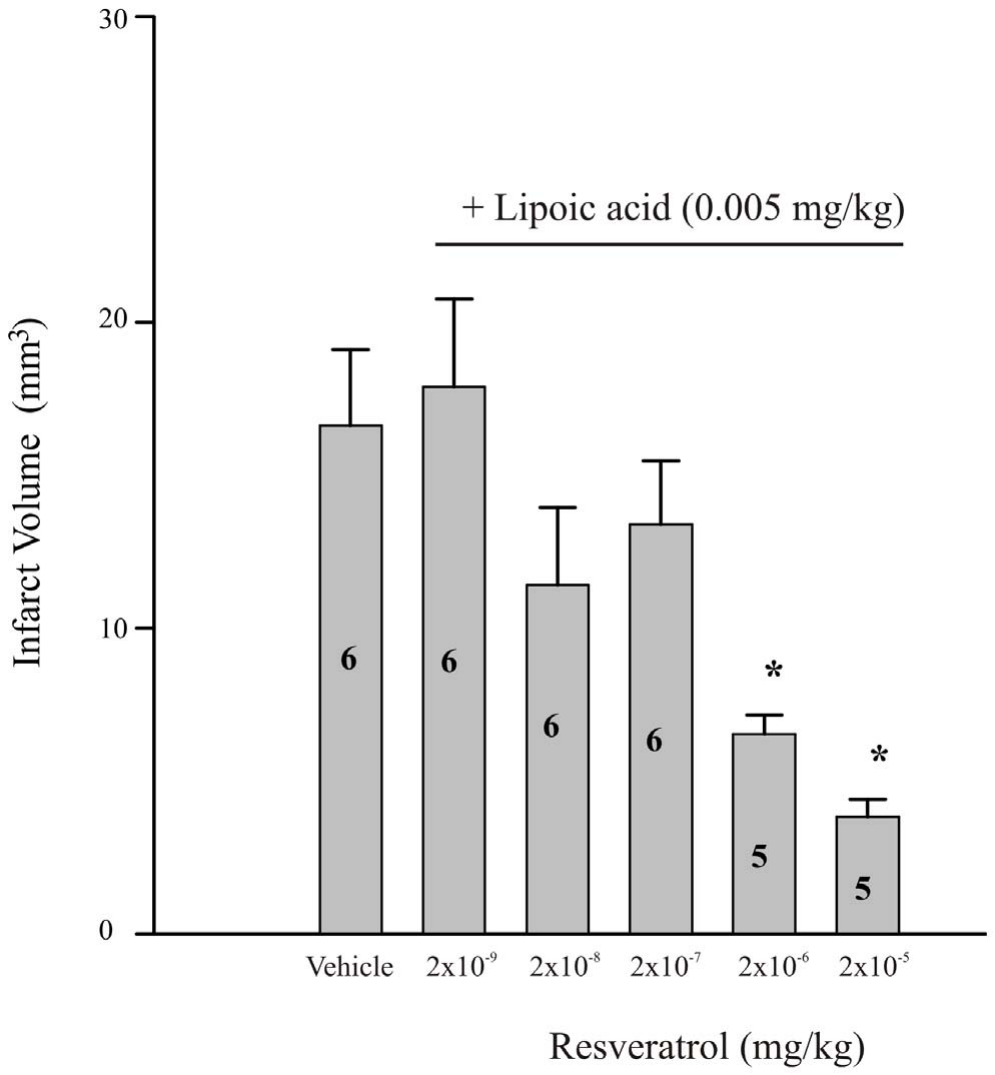
Effect of increasing doses of resveratrol in combination with lipoic acid on infarct volume. Bar graph summarizing the effect of co-administration of a sub-threshold dose of lipoic acid (0.005 mg/kg) with increasing doses of resveratrol on infarct volume following ischemia/reperfusion. Each bar represents the mean ± S.E.M. (n = 5–6/group) and * indicates significance (*p*≤0.05) from the vehicle-treated (propylene glycol 4×10^−3%^ (v/v)) control group.

**Figure 4:**
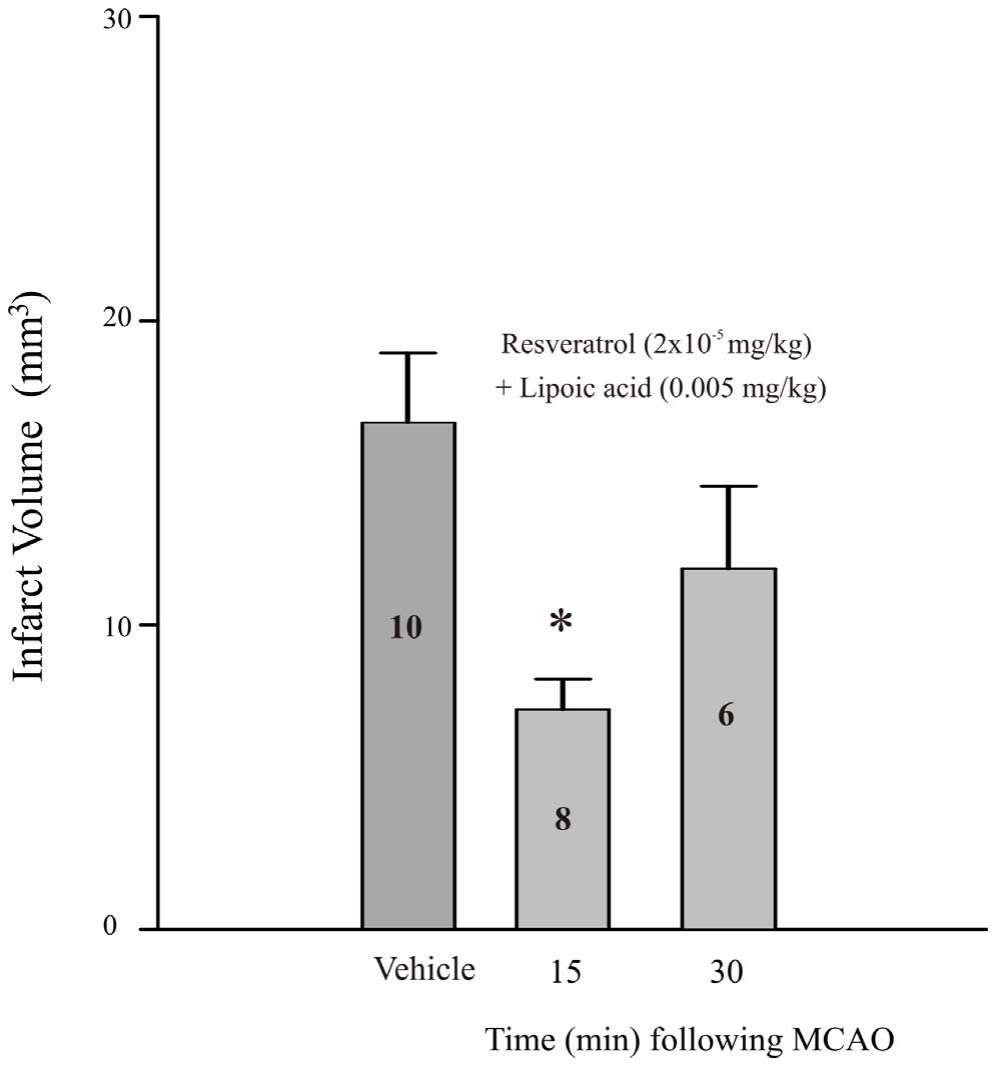
Time course of effect of combining lipoic acid and resveratrol on infarct volume. Bar graph summarizing the effect on infarct volume of administering a non-protective dose of lipoic acid in combination with a protective dose of resveratrol 15 minutes during the occlusion (15 min), or immediately after reperfusion (30 min). Each bar represents the mean ± S.E.M. (n = 5–8/group) and * indicates significance (*p*≤0.05) from the vehicle-treated (propylene glycol 4×10^−3%^ (v/v)) control group.

The combined pre-administration of resveratrol and LA 30 minutes prior to tMCAO produced a dose-dependent reduction in infarct volume compared to vehicle injected controls when measured following 5.5 hrs of reperfusion (Fig. 3). This effect was significant at the 2 highest doses of resveratrol (2×10^−6^ and 2×10^−5^ mg/kg; p≤0.05; Fig. 3). Delaying treatment of resveratrol (2×10^−5^ mg/kg) and LA (0.005 mg/kg) until 15 minutes following the onset of tMCAO was neuroprotective however no significant effect was observed when the same combination of resveratrol and LA was injected immediately prior to suture removal and the onset of reperfusion (30 minutes post occlusion; Fig. 4).

**Figure 5:**
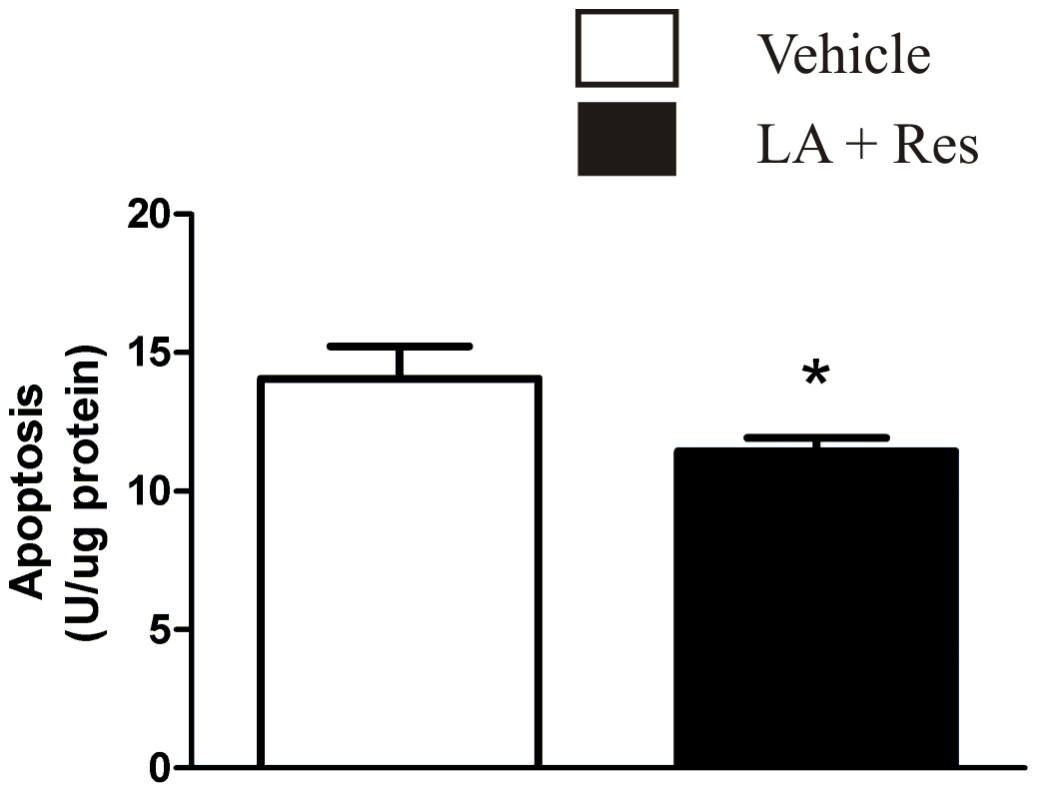
Effect of lipoic acid and resveratrol combination on a marker of apoptotic cell death. Bar graph of the quantified cytoplasmic histone-associated-DNA fragmentation (an indicator of apoptotic cell death) as obtained from a tissue sample (see methods for detailed description). Each bar represents the mean ± S.E.M. and * indicates significance (*p*≤0.05) from the vehicle-treated (propylene glycol 4×10^−3%^ (v/v)) control group.

Tissue sampled from the infarct region of rats injected with resveratrol (2×10^−5^ mg/kg) and LA (0.005 mg/kg) 30 minutes prior to tMCAO displayed lower levels of cytoplasmic histone-associated-DNA fragmentation. This biomarker suggests decreased apoptotic cell death (p≤0.05; Fig. 5).

Co-injection of resveratrol (2×10^−5^ mg/kg) and LA (0.005 mg/kg) 30 minutes prior to 6 hours of permanent MCAO did not produce significant neuroprotection (p≥0.05; data not shown). The average infarct volumes following 6 hrs of permanent MCAO in the vehicle and resveratrol - LA treated groups were 25.3±6 and 19.9±5 mm^3^ respectively.

### UPEI-200 and UPEI-201in tMCAO and pMCAO

**Figure 6:**
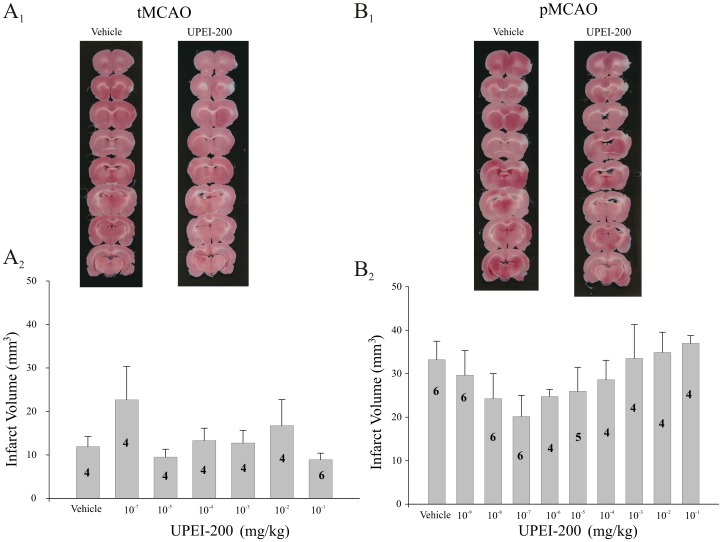
Lack of an effect of UPEI-200 on ischemic or reperfusion injury-induced cell death. (A_1_) Representative photomicrographs of TTC-stained sections from vehicle and UPEI-200-treated animals prior to either ischemia/reperfusion (tMCAO; A_1_) or permanent middle cerebral artery occlusion (6 hr pMCAO; B_1_). Bar graphs illustrating the lack of effect on infarct volume of UPEI-200 (3∶1 ratio of lipoic acid to resveratrol) at increasing doses or a vehicle (propylene glycol 4×10^−3%^ (v/v)) injected 30 minutes prior to either tMCAO (A_2_) or pMCAO (B_2_). Each bar represents the mean ± S.E.M. (n = 4–6/group).

UPEI-200 is a chemical construct composed of 3 LA moieties bonded to a single resveratrol molecule (3∶1). When administered 30 minutes prior to MCA occlusion in either tMCAO or pMCAO models, there was no significant neuroprotection observed at any of the doses tested (p≥0.05; Fig. 6A, 6B).

**Figure 7:**
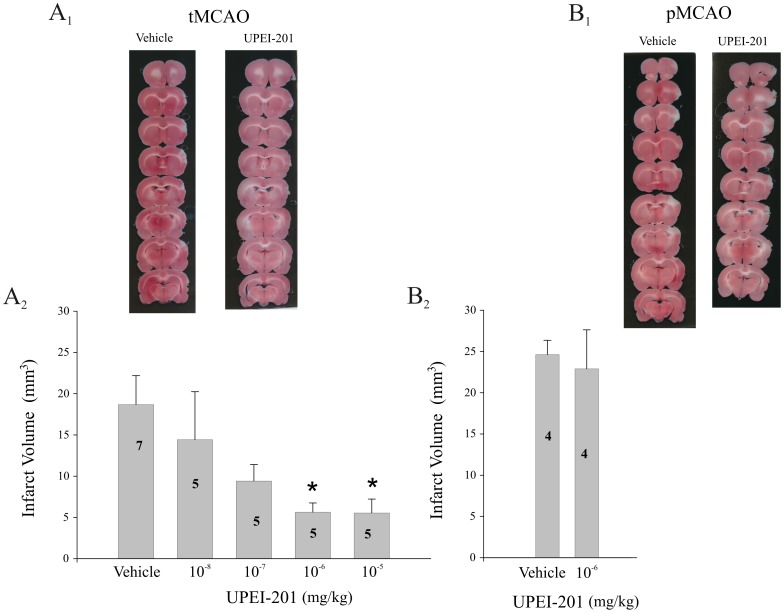
Dose-dependent effect of UPEI-201 on ischemic but not reperfusion injury-induced cell death. (A_1_) Representative photomicrographs of TTC-stained sections from vehicle and UPEI-201-treated animals prior to either ischemia/reperfusion (tMCAO; A_1_) or permanent middle cerebral artery occlusion (6 hr pMCAO; B_1_). Bar graph illustrating the effect on infarct volume of UPEI-201 (1∶1 ratio of lipoic acid to resveratrol) at increasing doses or a vehicle (propylene glycol 4×10^−3%^ (v/v)) injected 30 minutes prior to either ischemia/reperfusion (tMCAO; A_2_) or permanent middle cerebral artery occlusion (6 hr pMCAO; B_2_). Each bar represents the mean ± S.E.M. (n = 5–7/group) and * indicates significance (*p*≤0.05) from the vehicle-treated control group.

**Figure 8:**
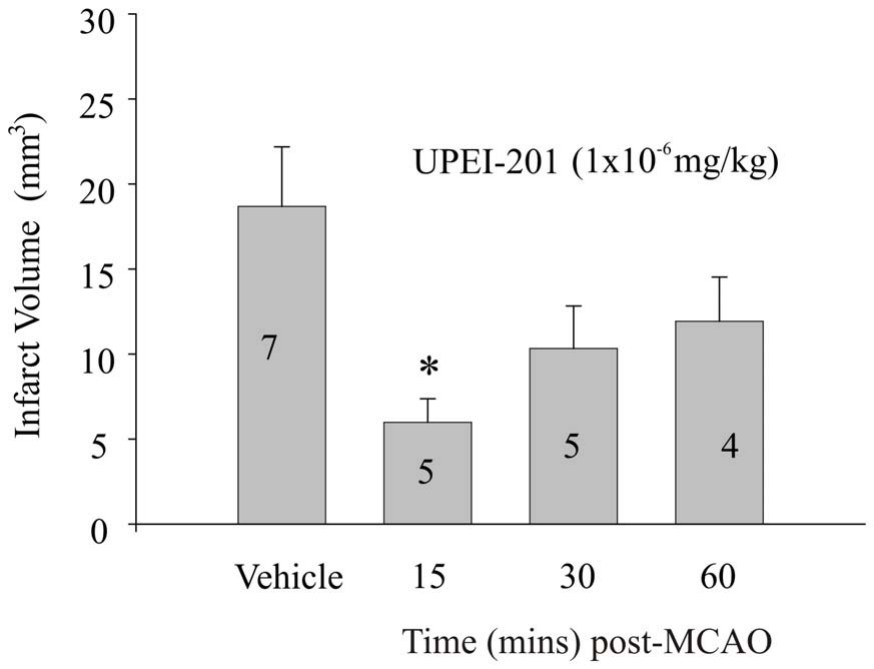
Time course of the effect of UPEI-201 on infarct volume. (A) Bar graph illustrating the effect on infarct volume of UPEI-201 (1∶1 ratio of lipoic acid to resveratrol) at a dose of 1×10^−6^ mg/kg or a vehicle (propylene glycol 4×10^−3%^ (v/v)) injected during the occlusion (15) or at 30 minute intervals immediately following reperfusion. Each bar represents the mean ± S.E.M. (n = 5–7/group) and * indicates significance (*p*≤0.05) from the vehicle-treated control group.

Conversely, UPEI-201, which is composed of a single LA moiety bound to resveratrol (1∶1), displayed potent neuroprotection when administered 30 minutes prior to MCA in tMCAO (Fig. 7A; p≤0.05). Delayed intervention with UPEI-201 (1×10^−6^ mg/kg) was successful in reducing infarct volume when administered 15 minutes into the occlusion period (15 min; p≤0.05, Fig. 8), but not when administered at the start or reperfusion or 30 minutes into the 5.5 hr reperfusion period (30, 60 min; Fig. 8).

### Effects of UPEI-201on Blood Pressure and Heart Rate

Since UPEI-201 was observed to provide neuroprotection in the tMCAO model, the following experiment was designed to determine the effect of UPEI-201 on arterial pressure and heart rate for a period of 2 hrs following administration. Baseline MAP and mean HR prior to drug administration were 109±9 mm/Hg and 378±27 bpm, respectively. Intravenous administration of UPEI-201 (1×10^−6^ mg/kg; n = 4) did not significantly alter mean arterial blood pressure or mean HR at any time point during the 2 hrs of continuous recording compared with vehicle (n = 4; P≥0.05; data not shown).

## Discussion

Dietary plant phenolics such as resveratrol are being widely used in supplement form to prevent and treat common health concerns. Potential safety issues exist as high doses of resveratrol have been shown to cause renal toxicity [25] and contribute to hepatic oxidative stress [26]. In the presence of peroxidase and/or transition metals, resveratrol may function as a pro-oxidant ultimately contributing to DNA damage and mitochondrial dysfunction [27] [28]. As well, resveratrol has been shown to inhibit cytochrome P450 enzyme CYP1A1 [29], an interference which may render other drugs in a patient’s treatment plan ineffective at therapeutic doses. Clearly, the health benefits of resveratrol are extensive and hence, finding ways to harness the potency of resveratrol in the absence of adverse side effects is desirable.

To this end, we show in this study that resveratrol on its own produced dose-dependent neuroprotection against neuronal cell death in a rodent model of transient ischemia-reperfusion injury [24]. Combined injection of resveratrol with a non-neuroprotective dose of α-lipoic acid [12] prior to tMCAO produced neuroprotection at doses of resveratrol 100 fold less than when injected alone. By chemically bonding resveratrol to lipoic acid in a 1∶1 ratio (UPEI-201), we were able to show a further dose reduction (ten-fold lower) coincident with significant neuroprotection which supports the advantage of combination therapy in stroke treatment.

Numerous studies have proven the efficacy of treatment with lipoic acid in disease states reflective of pro- and antioxidant imbalance such as diabetes, Alzheimer’s disease, cancer and cerebrovascular disease [30] [31] [32]. The chemical characteristics of LA, as well as its reduced form dihydrolipoic acid, qualify it as an effective scavenger of hydroxyl radicals, nitric oxide, peroxyl radicals and peroxynitrites, singlet oxygen species and hypochlorous acid [33]. Previously in our laboratory, we showed that LA pretreatment was effective as a neuroprotectant in both reperfusion injury following tMCAO [12] as well as in permanent ischemia (pMCAO) [13]. In the present study, combination of LA with resveratrol did not protect against neuronal death in a model of pMCAO. Prolonged ischemia is characterized by glutamate-induced neuronal toxicity ultimately leading to necrosis [3]. Generation of oxidative radicals is minimal owing to the lack of blood flow and dampening of mitochondrial activity, thereby rendering anti-oxidant therapy ineffectual. In contrast, anti-oxidants are highly effective in combating the oxidative stress generated during reperfusion injury which is demonstrated in the current study in our model of tMCAO. The reduction in infarct volume associated with resveratrol-LA treatment correlates with fewer necrotic cells at the ischemic core as evidenced with TTC staining, as well as reduced apoptotic cell death in the area of the penumbra as demonstrated by reduced oxidative DNA damage.

Other potential implications of combining resveratrol treatment with LA include their complementary participation in cell preserving pathways. For example, resveratrol and LA have both been shown to enhance aldehyde dehydrogenase-2-mediated detoxification of aldehydes in models of ethanol toxicity and ischemia-reperfusion injury respectively [4] [5]. Both compounds influence antioxidant status, in part through direct reduction of reactive oxygen species, but also as modulators of endogenous anti-oxidant systems. Resveratrol was shown to induce MnSOD activity in isolated rat liver mitochondria while LA inhibited glutathione peroxidase activity and induced mitochondrial uncoupling in the same model [34]. It is also noteworthy, that the LA/dihyrolipoate system is highly efficient in the reduction of the oxidized forms of antioxidants essentially aiding in their recycling allowing them to work more effectively without saturation [35]. Its dual solubility in water and lipid allows LA to interact with antioxidants in extracellular (blood) as well intracellular (both cytoplasmic and mitochondrial) compartments and to effectively cross the blood-brain barrier [36].

To utilize the strategy of combinatorial therapy, we created 2 new chemical entities, UPEI-200 and 201 and determined that a 1∶1 ratio of resveratrol-LA moieties (UPEI-201) was preferred in providing neuroprotection following ischemia-reperfusion (tMCAO). UPEI-201 effectively provided neuroprotection when injected 15 minutes into the period of occlusion but not when injected during reperfusion. With dosing in the nanomolar range providing significant neuroprotection in our model of transient ischemia, UPEI-201 is clearly a potent neuroprotectant against oxidative damage. Future studies will be necessary to address mechanism of action, bioactivation, plasma stability, and pre-conditioning applications. Recent figures estimate medication non-compliance at around 50% at a cost of $300 hundred billion a year [37]. Novel compounds such as UPEI-201 which aim to provide multi-level care in a single dose, may improve compliance and could make a significant contribution to global health initiatives in treating and/or preventing cerebrovascular disease. In contrast, the ineffectiveness of UPEI-200 to provide neuroprotection in either tMCAO or pMCAO paradigms clearly demonstrates the utility of bioassay-guided optimization to achieve the ideal ratio of newly synthesized bioactive molecules.

In conclusion, the results presented above support the notion that combining antioxidants at subthreshold doses can produce equal or enhanced neuroprotective effects. In addition, creation of novel chemical entities via the synthetic bonding of these antioxidants can produce comparable effects to those observed by the co-administration of the 2 compounds, but at lower doses. The clear advantage to lowering the dose required to gain therapeutic effect is to minimize off-target effects on other organ systems which may lead to side effects as is seen in so many of the prescription drugs on the market today.
